# Inconsistent approaches of the G-BA regarding acceptance of primary study endpoints as being relevant to patients - an analysis of three disease areas: oncological, metabolic, and infectious diseases

**DOI:** 10.1186/s12913-016-1902-8

**Published:** 2016-11-14

**Authors:** Thomas Staab, Georg Isbary, Volker E. Amelung, Jörg Ruof

**Affiliations:** 1 Roche Pharma AG, Emil-Barrell-Str. 1, 79639 Grenzach-Wyhlen, Germany; 2 Medical School of Hanover, Hanover, Germany

**Keywords:** Early benefit assessment, HTA, Marketing authorisation, Primary endpoint, AMNOG, Morbidity

## Abstract

**Background:**

Previous evaluations of oncological medicines in the German early benefit assessment (EBA) procedure have demonstrated inconsistent acceptance of endpoints by regulatory authorities and the Federal Joint Committee (G-BA). Accepted standard endpoints for regulatory purposes are frequently not considered as patient-relevant in the German EBA system.

In this study the acceptance of clinically acknowledged primary endpoints (PEPs) from regulatory trials in EBAs conducted by the G-BA was evaluated across three therapeutic areas.

**Methods:**

Medicines for oncological, metabolic and infectious diseases with EBAs finalised before 25 January 2016 were evaluated. Respective manufacturer’s dossiers, regulatory assessments, G-BA appraisals and oral hearing minutes were reviewed, and PEPs were examined to determine whether they were considered relevant to patients by the G-BA. Furthermore, the acceptance of symptomatic vs asymptomatic PEPs was also analysed.

**Results:**

A total of 65 EBAs were evaluated. Mortality PEPs were widely accepted as patient-relevant but were only used in a minority of EBAs and exclusively in oncological diseases. Morbidity PEPs constituted around 72 % of assessed PEPs, but were excluded from the EBA in over half of the corresponding assessments as they were not considered patient-relevant. Symptomatic endpoints were largely deemed patient-relevant, whereas acceptance of asymptomatic endpoints varied between therapeutic areas.

**Conclusions:**

This evaluation identified inconsistencies in patient relevance of morbidity-related PEPs as well as in acceptance of asymptomatic endpoints by the G-BA in all three disease areas examined. Better harmonisation between the regulatory authorities and the G-BA is still required after 5 years of AMNOG health technology assessment in Germany.

**Electronic supplementary material:**

The online version of this article (doi:10.1186/s12913-016-1902-8) contains supplementary material, which is available to authorized users.

## Background

Since 2011, all new medicines in Germany are required to undergo an early benefit assessment (EBA) in comparison to a pre-specified appropriate comparator [1]. At market entry, the pharmaceutical manufacturer (PM) needs to submit an EBA dossier evaluating the additional benefit of the drug, based on available clinical trial data, to the Federal Joint Committee (*Gemeinsamer Bundesausschuss*, G-BA).

The extent of additional benefit is classified by the G-BA as major (1), considerable (2), minor (3), non-quantifiable (4), no additional benefit (5), and less benefit (6) [2]. The benefit rating serves as the main basis for subsequent price negotiations for reimbursement between the PM and statutory health insurance providers.

For the determination of additional benefit the G-BA requests efficacy data in three different categories: mortality, morbidity and health-related quality of life (HRQoL) [2, 3]. However, the G-BA only takes into account endpoints deemed to be patient-relevant. Importantly, the opinion of the G-BA on patient relevance of endpoints frequently diverges from those of regulatory authorities [4–8].

A previous analysis of endpoints in oncology indicated acceptance by the G-BA of endpoints related to mortality, while morbidity endpoints were largely disregarded [4]. The current analysis extends the scope beyond oncology to evaluate if the identified trends also apply to other disease areas. Benefit assessments in the three most frequently assessed therapeutic areas (with ≥10 finalised and evaluable assessments) were analysed: oncological, metabolic, and infectious diseases. For all primary endpoints (PEPs), patient relevance was evaluated and used to determine whether these PEPs would be taken into consideration in an EBA by the G-BA.

## Methods

*Analysis set*: EBA dossiers submitted by the PMs, the corresponding G-BA appraisals, and oral hearing minutes, all obtained from the G-BA website, were used as sources of data [9]. Dossiers were not included in the analysis if the G-BA’s view on patient relevance of endpoints was not discernible. This could be due to i) no dossier being submitted by the PM, ii) inability to evaluate acceptance of PEP by the G-BA (due to use of inappropriate comparator, inadequate indirect comparison, or dossiers/studies being incomplete), or iii) if a more recent assessment for the same medicine and indication was available.

*Analysis of patient relevance of primary endpoints in the benefit assessment by the G*-*BA*: The resulting dataset was used to analyse whether PEPs used in the clinical studies and reported in the PM dossiers went on to be accepted as patient-relevant in the G-BA appraisals. PEPs were recorded as:

- *patient-relevant* when this was explicitly mentioned in the G-BA appraisal, or the corresponding data were used to justify an additional benefit,
- *partially patient-relevant* when the PEP was a composite or co-primary endpoint with both patient-relevant and non-patient-relevant components, or
- *not relevant to patients* when the G-BA clearly questioned the validity of the endpoint or the appraisal did not mention the PEP data or contained no clear statement of its inclusion.

Following determination of the G-BA’s view on patient relevance of each PEP, they were categorised as either symptomatic or asymptomatic. To ensure this categorisation of the endpoints was unbiased and meaningful, we utilised multiple sources of information integral to the EBA process to inform this procedure. Specifically, the categorisations were based on definitions and descriptions of the endpoints and the respective discussions within the dossiers, the assessments by the Institute for Quality and Efficiency in Healthcare (*Institut für Qualität und Wirtschaftlichkeit im Gesundheitswesen*, IQWiG), and the G-BA appraisals. The oral hearing at the G-BA aims to provide clarification on critical and controversial topics. Key representatives of the relevant medical and scientific societies are always attending those hearings. Thus, the oral hearing minutes served as a source of expert opinion which we referred to for categorisation of certain endpoints along with supporting scientific statements.

The medicines were divided into therapeutic areas, as categorised by the G-BA, and only therapeutic areas with ≥10 finalised and evaluable EBAs as of 25 January 2016 were considered, which led to the inclusion of oncological, metabolic and infectious diseases in the final analysis set.

## Results

### Analysis set

Inclusion of therapeutic areas with ≥10 finalised and evaluable EBAs (oncological, metabolic, and infectious disease areas) resulted in 97 EBAs which were considered for evaluation. Of these, 65 (67 %) were evaluable according to the inclusion criteria. Table 1 summarises the number of EBAs included in the analysis and the main reasons for exclusion of EBAs from the analysis set by therapeutic area.

**Table 1:** Inclusion vs exclusion of EBAs by therapeutic area

Therapeutic area	EBAs (*n*)	Assessments in analysis set (*n*)		Main reason for EBAs not being evaluable^a^		
		Included	Excluded	No dossier submitted	Data on PEP acceptance by G-BA not evaluable	Newer assessment available (same medicine and indication)
Oncological diseases	47	36	11	2	6	3
Metabolic diseases	34	15	19	3	14	2
Infectious diseases	16	14	2	0	2	0
Total	97	65	32	5	22	5

^a^EBAs were excluded from the analysis when no dossier was submitted by the PM, a newer assessment was available (same medicine and indication) or the G-BA’s viewpoint on the patient relevance of the PEP could not be discerned (due to incomplete dossiers or studies, use of inappropriate comparators, or inadequate indirect comparisons)

*EBA* Early benefit assessment, *G-BA* Federal Joint Committee, *n* Number of EBAs, *PEP* Primary endpoint, *PM* Pharmaceutical manufacturer

A high proportion of EBAs in metabolic diseases (19 of 34, 56 %) were not evaluable, mainly because the G-BA objected to the methodological execution of the studies for reasons including use of inappropriate comparator or indirect comparison, and incomplete dossiers or studies. A list of EBAs and the respective PEPs evaluated in the analysis is presented in Table 2.

**Table 2:** EBAs included in the analysis set and respective primary endpoints

Medicine	Indication	Primary endpoint (PEP)	Therapeutic expert panel present at oral hearing
Oncological diseases			
Abiraterone acetate	Prostate carcinoma	OS	BDU, DGHO, DVPZ
Abiraterone acetate^a^	Prostate carcinoma	OS, rPFS	DGHO
Afatinib^c^	Non-small cell lung cancer	PFS	DGHO, DKG (Working Group on Thoracic Oncology of the AIO)
Aflibercept	Metastatic colorectal cancer	OS	DGVS
Axitinib	Renal cell carcinoma	PFS	DGHO
Bosutinib	Chronic myeloid leukaemia	MCR	DGHO
Brentuximab vedotin	Hodgkin-Lymphoma, anaplastic large cell lymphoma	ORR	DGHO
Cabazitaxel	Prostate carcinoma	OS	DGHO, DKG
Cabozantinib	Thyroid gland neoplasia	PFS	DGE (thyroid gland section), DGHO, DGN
Crizotinib	Non-small cell lung cancer	PFS, ORR	DGHO, POA, representatives from leading oncology centres
Decitabine	Myeloid leukaemia	OS	DGHO
Enzalutamide	Prostate carcinoma	OS	DGHO, DGU
Enzalutamide^a^	Prostate carcinoma	OS, rPFS	DGHO
Eribulin	Breast cancer	OS	DGHO
Eribulin^ac^	Breast cancer	OS, rPFS	DGHO
Ibrutinib	Chronic lymphocytic leukaemia, relapsed or refractory mantle cell lymphoma (MCL)	ORR	DGHO
Idelalisib	Chronic lymphocytic leukaemia, follicular lymphoma	PFS	DGHO, GLSG, representative from the University Hospital Gießen
Ipilimumab	Melanoma	OS	DGHO
Lenvatinib	Thyroid gland neoplasia	PFS	DGHO
Nintedanib	Non-small cell lung cancer	PFS	DGHO, DKG (Working Group on Thoracic Oncology of the AIO), representative from the LungenClinic Grosshansdorf
Nivolumab	Melanoma	OS, PFS	ADO, DGHO
Obinutuzumab	Chronic lymphocytic leukaemia	PFS	DGHO
Olaparib	Ovarian cancer	PFS	DGHO
Pertuzumab	Breast cancer	PFS, ORR	AGO, DGHO
Pomalidomide	Multiple myeloma	PFS	DGHO, representatives from the University Hospitals of Heidelberg, Tübingen and Würzburg
Radium-223-dichloride	Prostate carcinoma	OS	DGHO
Ramucirumab	Stomach cancer	OS	DGHO, DGVS
Regorafenib	Colorectal cancer	OS	DGHO
Ruxolitinib^d^	Chronic myeloproliferative diseases	≥35 % reduction in spleen volume	DGHO
Ruxolitinib^a^	Polycythaemia vera	Haematocrit control without phlebotomy and ≥35 % reduction in spleen volume	DGHO
Siltuximab	Multicentric Castleman’s disease	Durable tumour & symptomatic response (complete and partial response)	DGHO
Sipuleucel-T	Prostate carcinoma	OS	DGHO, representative from the University Hospital Tübingen, Department of Urology
Trastuzumab emtansine	Breast cancer	OS, PFS	AGO, DGHO
Vandetanib^b^	Thyroid gland neoplasia	PFS	DGHO
Vemurafenib^c^	Melanoma	OS, PFS	ADO, DGHO
Vismodegib	Basal cell cancer	ORR	DGHO, DGMKG, German Society of Dermatology
Metabolic diseases			
Albiglutide	Diabetes mellitus type 2	HbA1c	DDG, Diabetes Research Group HZM, representative from the University Hospital Carl Gustav Carus (Dresden)
Dulaglutide	Diabetes mellitus type 2	HbA1c	BVND, DDG, Diabetes Research Group HZM
Eliglustat	Gaucher disease type 1	Stable health status (decrease in spleen and liver volume, Hb, thrombocytes) and %-change in spleen volume	ASIM, DGVS, representative from the Charité University Medicine Berlin
Elosulfase alfa	Mucopolysaccharidose type IVA (Morquio A syndrome)	6MWT	ZSE Wiesbaden
Insulin degludec^a^	Diabetes mellitus type 1	HbA1c	DDG
Ivacaftor	Cystic fibrosis	FEV1%	-
Ivacaftor^a^	Cystic fibrosis	FEV1%	-
Linagliptin^b^	Diabetes mellitus type 2	HbA1c	BVND, DDG, Diabetes Research Group HZM
Pasireotide	Pituitary gland dysfunction	mUFC ≤ ULN	-
Pasireotide^a^	Acromegaly	Biochemical control	-
Saxagliptin	Diabetes mellitus type 2	HbA1c	DDG, Working Group on Pharmacoepidemiology, Diabetes Research Group HZM, Diabetes Centre Bad Lauterberg
Saxagliptin/metformin	Diabetes mellitus type 2	HbA1c	BVND
Sitagliptin	Diabetes mellitus type 2	HbA1c	DDG, Working Group on Pharmacoepidemiology, Diabetes Research Group HZM, Diabetes Centre Bad Lauterberg
Sitagliptin/metformin	Diabetes mellitus type 2	HbA1c	DDG, Diabetes Centre Bad Lauterberg, Diabetes Research Group HZM, Working Group on Pharmacoepidemiology
Vildagliptin^b^	Diabetes mellitus type 2	HbA1c	BVND, DDG
Infectious diseases			
Boceprevir	Chronic hepatitis C	SVR	bng, DGVS, German Liver Foundation
Daclatasvir	Chronic hepatitis C	SVR	bng, dagnä, DGIM, DGVS
Dasabuvir	Chronic hepatitis C	SVR	bng, dagnä, DGIM, DGVS
Dolutegravir	HIV infection	VR	dagnä, DAIG
Dolutegravir/abacavir/lamivudine	HIV infection	VR	-
Elvitegravir/cobicistat/emtricitabin/tenofovir-disoproxil	HIV infection	VR	dagnä, DAIG
Emtricitabine/rilpivirine/tenofovirdisoproxil	HIV infection	VR	dagnä, DAIG
Fidaxomicin	Clostridium infection	Overall cure	DGHO, DGVS
Ledipasvir/sofosbuvir	Chronic hepatitis C	SVR	bng, dagnä, DGIM, DGVS
Ombitasvir/paritaprevir/ritonavir	Chronic hepatitis C	SVR	bng, dagnä, DGIM, DGVS
Rilpivirine	HIV infection	VR	DAIG, dagnä
Simeprevir	Chronic hepatitis C	SVR	bng, dagnä, DGI, DGIM, DGVS
Sofosbuvir	Chronic hepatitis C	SVR	bng, dagnä, DGIM
Telaprevir	Hepatitis C	SVR	bng, DGVS, German Liver Foundation

^a^New therapeutic indication, ^b^Re-assessment for the same indication, ^c^Re-assessment after expiration of G-BA appraisal, ^d^Ruxolitinib is an orphan drug, but has undergone a regular EBA process after having reached sales of >€50 million per year

*6MWT* 6-minute walking test, *ADO* Working Group on Dermatological Oncology, *AGO* Working Group on Gynaecologic Oncology, *AIO* Working Group on Internal Oncology, *ASIM* Working Group on Congenital Metabolic Disorders in Internal Medicine, *BDU* Professional Association of German Urologists, *bng* Federal Association of Registered Gastroenterologists, *BVND* Federal Association of Registered Diabetologists, *dagnä* German Working Group of Registered Doctors in the Care of HIV-infected Persons, *DAIG* German AIDS Society, *DDG* German Diabetes Society, *DGE* German Society of Endocrinology, *DGHO* German Society of Hematology and Medical Oncology, *DGI* German Society of Infectious Diseases, *DGIM* German Society of Internal Medicine, *DGMKG* German Society of Oral and Maxillofacial Surgery, *DGN* German Society of Nuclear Medicine, *DGU* German Society of Urology, *DGVS* German Society of Gastroenterology, Digestive and Metabolic Diseases, *DKG* German Cancer Society, *DVPZ* Umbrella Organisation of Prostate Centres in Germany, *EBA* Early benefit assessment, *FEV1* Forced expiratory volume in 1 second, *GLSG* German Low Grade Lymphoma Study Group, *Hb* Haemoglobin, *HbA1c* Glycated haemoglobin, *HIV* Human immunodeficiency virus, *HZM* Helmholtz Centre Munich, *POA* Working Group on Pulmonary Oncology, *MCR* Major cytogenic response, *mUFC* Median urinary free cortisol, *ORR* Objective response rate, *OS* Overall survival, *PFS* Progression-free survival, *rPFS* Radiographic progression-free survival, *SVR* Sustained viral response, *ULN* Upper limit of normal, *VR* Viral response, *ZSE* Centre for Rare Diseases

### Analysis of patient relevance of primary endpoints in the benefit assessment by G-BA

#### All therapeutic areas

This analysis set consisted of 65 PEPs obtained from 65 EBA dossiers. The G-BA deemed the PEPs as relevant to patients in 31 (48 %) of 65 EBAs, not relevant to patients in 25 (38 %), and partially patient-relevant in nine (14 %) assessments (refer to sections on individual therapeutic areas for more details on specific PEPs).

**Fig. 1:**
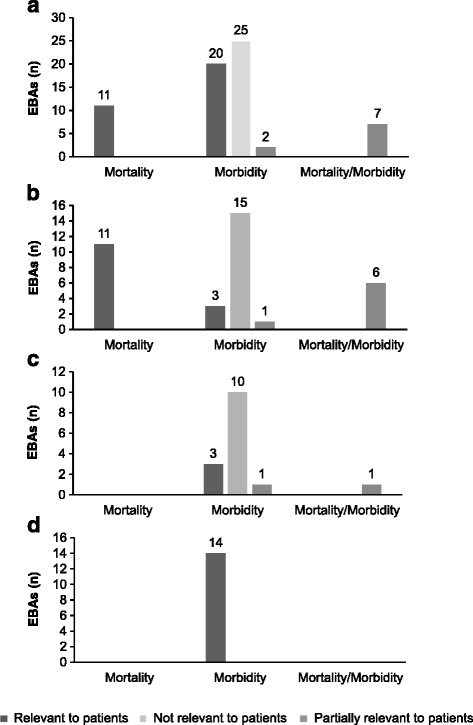
Patient relevance of PEPs according to the G-BA by endpoint dimension. **a** All three therapeutic areas, **b** oncological diseases, **c** metabolic diseases, and **d** infectious diseases. HRQoL was not used as PEP for any assessment, therefore it is not shown in the graphs

Analysis by endpoint category showed that the majority of PEPs were morbidity endpoints (47 of 65, 72 %). Mortality PEPs were used in 11 of 65 PM submissions (17 %), all of which concerned oncological diseases and were deemed patient-relevant by the G-BA (Fig. 1a). Morbidity PEPs were considered patient-relevant in 20 of 47 (43 %) EBAs and partially patient-relevant in two cases (4 %). In seven EBAs the PEP was a composite endpoint of mortality and morbidity and in each case deemed partially patient-relevant. Six of these cases were in oncological diseases and one was in the metabolic disease area (see also sections on individual therapeutic areas). No assessment included HRQoL as a PEP.

#### Oncological diseases

In oncological diseases 36 of 47 PM submissions were analysed (Table 1). Mortality PEPs were reported in 11 of the 36 EBAs (all overall survival [OS]) and regarded as patient-relevant in the benefit assessment by the G-BA in all cases (Fig. 1b). Fifteen of 19 (79 %) morbidity PEPs (mostly progression-free survival [PFS]) were deemed not patient-relevant. The three patient-relevant morbidity PEPs were major cytogenic response (MCR) (bosutinib, chronic myeloid leukaemia), reduction of spleen volume by >35 % (ruxolitinib, chronic myeloproliferative diseases), and haematocrit control without phlebotomy and reduction of spleen volume by >35 % (ruxolitinib, polycythaemia vera). For one composite morbidity endpoint, namely partial and complete durable tumour and symptomatic response, only complete response was considered patient-relevant and included in the G-BA appraisal (siltuximab, multicentric Castleman’s disease). Six assessments had co-primary endpoints, comprising mortality and morbidity (all OS and PFS). In all these cases only OS was considered in the appraisal.

#### Metabolic diseases

In metabolic diseases, 34 PM submissions were available, 15 of which were analysed (Table 1). All PEPs were morbidity endpoints, apart from one composite mortality/morbidity PEP (Fig. 1c) and three morbidity PEPs were deemed patient-relevant in the benefit assessments. One of these PEPs was glycated haemoglobin (HbA1c) in type 1 diabetes (insulin degludec), which was explicitly specified by the G-BA as a validated and patient-relevant endpoint. The others were 6-minute walk test (6MWT) in type IVA mucopolysaccharidose (elosulfase alfa), and median urinary free cortisol level (mUFC) in pituitary gland dysfunction (pasireotide).

In the ten EBAs where PEPs were not considered patient-relevant by the G-BA, PEPs were HbA1c and FEV1 (forced expiratory volume in 1 second) in type 2 diabetes and cystic fibrosis, respectively. In eight benefit assessments (albiglutide, dulaglutide, linagliptin [re-assessment], saxagliptin, saxagliptin/metformin, sitagliptin, sitagliptin/metformin, vildagliptin [re-assessment]) the PEP HbA1c was not explicitly mentioned as patient-relevant and not considered in the G-BA benefit evaluation, and therefore assumed to be not relevant to patients according to the evaluation criteria of this analysis stated above. In the two assessments of ivacaftor, FEV1 data was not taken into consideration by the G-BA as there were different opinions within the G-BA regarding its patient relevance.

In two EBAs the PEP was only partially accepted as patient-relevant by the G-BA. For eliglustat (Gaucher disease type 1) the PEP was a co-primary endpoint, consisting of two endpoints. The first was the composite morbidity endpoint stable health status (spleen volume, liver volume, haemoglobin and thrombocyte count) and the second percentage change in spleen volume, where only the change in spleen volume was deemed patient-relevant. For pasireotid (acromegaly) the PEP was biochemical control (defined as a combination of reduction of the mean growth hormone [GH] level below 2.5 μg/l and normalisation of the age- and sex-adjusted insulin-like growth factor 1 [IGF-1] level after 24 weeks). The PM defined biochemical control as a composite mortality/morbidity endpoint. However the G-BA did not accept the mortality component due to lack of validation.

#### Infectious diseases

In infectious diseases 14 of 16 PM submissions were included in the analysis set (Table 1). There were no mortality PEPs in infectious diseases. All 14 PEPs concerned morbidity (Fig. 1d), and all were accepted as patient-relevant by the G-BA. In most cases (13 of 14) the PEP was viral response (sustained viral response [SVR] in hepatitis C or viral response [VR] in HIV-infected patients), and in one case, the PEP was overall cure (fidaxomicin, clostridium infection).

### Acceptance of symptomatic vs asymptomatic PEPs

PEPs were categorised as symptomatic or asymptomatic according to the definition of the PEP and based on the appraisal documents as well as scientific discussions during the hearing process. A list of therapeutic area expert panels attending the oral hearings is shown in Table 2. Table 3 summarises each evaluated PEP and the results of the categorisation by therapeutic area (see Additional file 1: Table S1 for a table listing the rationale of categorisation for each endpoint).

**Table 3:** G-BA acceptance of symptomatic vs asymptomatic morbidity PEPs as (A) patient-relevant and (B) non-patient-relevant

	Oncological diseases	Metabolic diseases	Infectious diseases
(A) Morbidity PEPs accepted as patient-relevant by the G-BA			
Symptomatic	Complete durable tumour & symptomatic response^a^	6MWT	Overall cure
	≥35 % reduction in spleen volume^a^	Reduction in spleen volume^a^	-
Asymptomatic	Haematocrit control without phlebotomy^a^	HbA1c (Type 1 diabetes)	Viral response (VR, SVR)
	MCR	mUFC	-
	-	Biochemical control (mean GH <2.5 μg/L and normalisation of IGF-1)	-
(B) Morbidity PEPs not accepted as patient-relevant by the G-BA			
Symptomatic	Partial durable tumour & symptomatic response^a^	-	-
Asymptomatic	PFS^a^	HbA1c (Type 2 diabetes)	-
	ORR	Haemoglobin level^a^	-
	-	Thrombocyte count^a^	-
	-	Reduction in liver volume^a^	-
	-	FEV1	-

^a^This PEP was a component of a co-primary endpoint in at least one dossier

*6MWT* 6-minute walking test, *FEV1* Forced expiratory volume in 1 second, *G-BA* Federal Joint Committee, *GH* Growth hormone, *HbA1c* Glycated haemoglobin, *IGF-1* Insulin-like growth factor 1, *MCR* Major cytogenic response, *mUFC* Median urinary free cortisol, *ORR* Objective response rate, *OS* Overall survival, *PEP* Primary endpoint, *PFS* Progression-free survival, *SVR* Sustained viral response, *VR* Viral response

Asymptomatic PEPs mostly comprised laboratory parameters (e.g. HbA1c in diabetes) or endpoints involving imaging outcomes (e.g. PFS or objective response rate [ORR]) [9]. Symptomatic endpoints included outcomes directly experienced by the patient, for example OS in oncological conditions and 6MWT in mucopolysaccharidose [9]. For other PEPs (complete/partial durable tumour and symptomatic response, overall cure), symptomaticity was self-explanatory by their definitions [9]. Regarding the symptomaticity of the PEPs related to reduction in spleen or liver volume, an independent Gaucher disease type 1 expert (a member of the German Working Group for Congenital Metabolic Disorders in Internal Medicine [*Arbeitsgemeinschaft für angeborene Stoffwechselstörungen in der Inneren Medizin*, ASIM]) stated in the minutes of the oral hearing procedure for eliglustat that the spleen volume in patients prior to treatment is typically increased 15-fold to ca. 2 L [9]. Since the measured reduction with eliglustat treatment was around 30 % and therefore easily palpable we assigned the reduction in spleen volume as a symptomatic endpoint. A similar reasoning applies to the reduction of ≥35 % of spleen volume in the EBA of ruxolitinib in the indications chronic myeloproliferative diseases and polycythaemia vera. In contrast, a closer analysis of the data for Gaucher disease showed that the relative enlargement and reduction in liver volume before and after eliglustat treatment were too small to be palpable and thus deemed not symptomatic.

Subsequently, we compared the consideration of symptomatic vs asymptomatic PEPs by the G-BA in evaluating additional benefit. For three PEPs the oral hearing minutes were consulted in order to categorise them as symptomatic or asymptomatic, as described above. Symptomatic endpoints were mostly regarded as patient-relevant (Table 3 A), apart from partial durable tumour and symptomatic response (siltuximab, multicentric Castleman’s disease) (Table 3 B). Asymptomatic endpoints (mainly laboratory parameters and endpoints assessed by imaging techniques) were largely deemed not relevant to patients. This had considerable impact on benefit assessment of oncology medications, since PFS and ORR are widely used PEPs which are generally accepted as patient-relevant by the European Medicines Agency (EMA), but were not deemed patient-relevant by the G-BA (Table 3 B). In metabolic diseases HbA1c was only considered patient-relevant in type 1 diabetes. In contrast, asymptomatic viral response endpoints (VR and SVR) in infectious diseases (mainly hepatitis C and HIV infection) were readily accepted as patient-relevant by the G-BA.

## Discussion

This analysis demonstrates that specific morbidity PEPs from the EBA are categorically excluded by the G-BA across major disease areas, irrespective of indication and disease stage. Typically, these are PEPs used in pivotal studies for marketing authorisation. The respective clinical studies were specifically designed to show clinical benefit based on these endpoints. The aim of the EBA is to assess the additional benefit of a new medicine compared to the prespecified comparative treatment. This generally differs from the aim of regulatory authorities, which is to evaluate the benefit vs risk profile of a new medicine. Nevertheless, the methods and standards of evidence-based medicine apply to both evaluation procedures. Furthermore, primary endpoints of pivotal studies which led to EMA approval of the investigated drug should be accepted as patient-relevant, irrespective of whether ‘benefit’ or ‘additional benefit’ is under consideration. To suggest that these primary study endpoints are not relevant to patients, as the G-BA does, creates a major dilemma in clinical development and implicitly questions the ethical conduct of studies.

Our analysis by outcome dimension showed a strong dominance of morbidity over mortality PEPs (47 vs 11). While the mortality PEPs were accepted as patient-relevant without exception, less than half of the morbidity PEPs were deemed patient-relevant. Interestingly, the acceptance varied by therapeutic area, revealing a disadvantage for oncological and metabolic disease indications, where morbidity PEPs were predominantly regarded as not patient-relevant. Considering that the majority of these PEPs were accepted by regulatory authorities for marketing authorisation, the classification of such a large proportion of PEPs as not relevant to patients by the G-BA highlights marked differences in data interpretation by regulatory and health technology assessment (HTA) bodies. A similar finding was also demonstrated in a comparative analysis of parallel scientific advice of different European HTA bodies and the EMA [10].

The PEPs that were most frequently dismissed as not patient-relevant were PFS in oncological diseases and HbA1c in metabolic diseases (except for type 1 diabetes). Despite opposing views on patient relevance of PFS within the G-BA (e.g. afatinib, axitinib, crizotinib, eribuline) [9] data on this endpoint were systematically excluded from benefit assessments, based on the justification that PFS evaluation utilises asymptomatic endpoint assessment techniques such as imaging. Conversely, in infectious diseases, the PEP SVR was accepted as patient-relevant in hepatitis C despite being an asymptomatic surrogate endpoint lacking formal validation. PEPs in neurological diseases were also evaluated but ultimately excluded from the analysis set as only six assessments were evaluable. Here, morbidity PEPs were largely accepted as patient-relevant by the G-BA (5 of 6 EBAs).

The results of our analysis clearly demonstrate inconsistency in the G-BA’s approach to judging the patient relevance of PEPs between disease areas. For example, in infectious diseases, the G-BA has shown some flexibility in accepting the asymptomatic endpoint SVR as patient-relevant. On the other hand, in oncological and metabolic diseases some asymptomatic endpoints (PFS in cancers and HbA1c in type 2 diabetes) are categorically dismissed, without taking into consideration the different disease profiles of the indications. This is in contrast to the EMA, which adopts a broader approach when evaluating patient relevance and takes indication, difficulty in obtaining mortality data, and priority of accelerating patient access into account.

We welcome the flexibility the G-BA has shown regarding the acceptance of SVR as patient-relevant in hepatitis C, but identify a need to expand this flexibility to other endpoints. The general dismissal of PFS is questionable since in some cancers, such as ovarian cancer, it has been suggested that improved PFS is associated with clinical benefit and is a valid surrogate for extended OS [11]. Nevertheless the G-BA did not accept PFS as patient-relevant in the EBA of olaparib for the treatment of ovarian cancer [9].

The success of novel anticancer drugs in recent years has led to a classification shift of various oncological conditions from acutely fatal to chronic disease. Similar to chronic diseases such as diabetes, it is challenging to obtain mortality data showing significant differences in those oncological diseases with slow progression and low death rates. For these reasons, PFS has gained importance as a PEP in clinical trials because it is an early detectable and meaningful endpoint for disease progression [12, 13].

Similarly to PFS, HbA1c is routinely used as a surrogate endpoint in diabetes mellitus and widely accepted by regulatory authorities [14]. For example the EMA states in its “Note for guidance on clinical investigation of medicinal products in the treatment of diabetes mellitus” that HbA1c is the most widely accepted measure of overall, long-term blood glucose control in type 1 and type 2 diabetes, and therefore an appropriate primary study endpoint [15]. Moreover, this guidance states that reduction of HbA1c is directly related to a reduced risk of developing vascular complications. The G-BA, however, only accepts HbA1c as a patient-relevant endpoint in type 1 diabetes, but not in type 2.

For the patient relevance of morbidity endpoints, clarification of its definition and determination criteria by the G-BA are urgently needed, particularly in oncological diseases and type 2 diabetes, as currently no clear criteria for patient relevance are listed in the G-BA rules of procedure [2]. Representatives from patient advocacy groups and other external experts are consulted at several stages of the assessment procedure, potentially also concerning the patient relevance of endpoints, but their influence on the benefit appraisal is not transparent and has been criticised as insufficient [16, 17]. In a recent press release from the German Society for Hematology and Medical Oncology (*Deutsche Gesellschaft für Hämatologie und Medizinische Onkologie*, DGHO) a DGHO board member criticised the fact that market authorisation, EBA, and treatment guidelines often come to different conclusions, despite being based on the same clinical data, making it difficult for doctors to make treatment decisions [18].

Alignment of study requirements between the G-BA and regulatory authorities is necessary in order to optimise trial design and reduce patient sample sizes, particularly in rare indications, in order to allow timely access to new treatments. A recent publication has identified considerable heterogeneity in regulatory and HTA approaches, even among different European HTA bodies [10]. Regulatory authorities have adapted their approval pathways for innovative and promising new medicines to facilitate early patient access to new treatments, for example via conditional approval or adaptive pathways [10]. In order to effectively establish early access to medicines, HTA procedures need to follow the EMA’s footsteps and provide harmonised, transparent, flexible, conditional, and adaptive methods that adopt the level of evidence accepted by medicines agencies [10].

The UK National Institute for Health and Clinical Excellence (NICE) uses a less restrictive approach in their HTA procedure, where appropriate methods may be used to extrapolate data from less than ideal study designs regarding study type, study duration, patient population, choice of comparator, and type of outcomes [19]. In contrast to the approach adopted by the G-BA, where data that do not fully comply with requirements are largely rejected, NICE is more receptive to data derived from clinical trials which are for instance non-randomised, non-comparative or employ modelling.

Our analysis has revealed that asymptomatic endpoints were frequently disregarded by the G-BA, while symptomatic endpoints were largely deemed patient-relevant. However, acceptance of asymptomatic endpoints varied by therapeutic area. A major achievement in modern medicine is the ability to capture aspects of morbidity prior to their transformation in symptomatic disease in diagnostic and prognostic examinations. For example, in the assessment of axitinib for renal cell carcinoma, PFS was used to assess the delay in onset of metastases by imaging [9]. It is evident that early detection of metastases before the onset of symptomatic pain or even vertebral fractures is essential and of utmost relevance to the patient. In this case, the strict insistence on symptomatic endpoints may lead to a delay in complementary treatment options, such as bisphosphonates, which could prevent occurrence of vertebral fractures [20]. This would contradict the underlying ethical principles of diagnosis and therapy in this and many other oncological diseases.

**Fig. 2:**
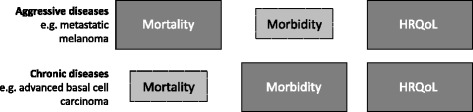
Adaptable and disease-specific weighting of endpoint categories

Currently, the G-BA demands patient-relevant outcome data for the three endpoint categories mortality, morbidity and HRQoL, and weighs each with equal importance for all assessments. However, a number of reasons may lead to attenuation of mortality data, for instance, i) clinical advancement of available therapies, ii) change in disease status from ‘untreatable’ to ‘chronic’, and iii) differences in the course of diseases themselves. Given the differences in mortality and progression between different diseases, for example some forms of cancer vs non-fatal chronic diseases, equal weighting of endpoint categories is not informative. Instead, differential weighting of each endpoint category in the EBA, taking into account disease type and severity/stage, as outlined in Fig. 2 would be advisable.

### Limitation

For each EBA evaluated, only the respective PEP was included, as this is the parameter which provides the most clinically relevant and convincing evidence, based on the statistical principles of clinical trials [21], and clinical trials are explicitly designed to show efficacy in terms of the PEP. A more comprehensive approach would also include secondary endpoints. Nevertheless, our approach sufficiently highlights the clear inconsistencies that exist between endpoint acceptance by regulatory authorities and the G-BA.

## Conclusions

There is a need for the G-BA to define the morbidity endpoints that are considered patient-relevant, particularly in indications such as diabetes and oncological diseases. This will allow PMs to target clinical trial designs towards requirements of the G-BA at an early clinical development stage to ensure an efficient pipeline for bringing innovative and efficacious treatments to patients. In addition, further harmonisation between regulatory bodies and G-BA with regards to acceptance of PEPs as relevant to patients is urgently required. In particular, this applies to morbidity-related PEPs that are not (yet) symptomatic and tangible to patients.

## Additional file

Additional file 1: Table S1. Rationale of categorisation of PEPs as symptomatic and asymptomatic. List of the rationale of categorisation as symptomatic and asymptomatic for all PEPs included in the analysis. (DOCX 14 kb)

## Abbreviations

6MWT: 6-minute walking test
ADO: Working Group of Dermatological Oncology
AGO: Working Group of Gynaecological Oncology
AIO: Working Group of Internal Oncology
AMNOG: The Act on the Reform of the Market for Medicinal Products
ASIM: Working Group for Congenital Metabolic Disorders in Internal Medicine
BDU: Professional Association of German Urologists
bng: Federal Association of Registered Gastroenterologists
BVND: Federal Association of Registered Diabetologists
dagnä: German Working Group of Registered Doctors in the Care of HIV-infected Persons
DAIG: German AIDS Society
DDG: German Diabetes Society
DGE: German Society of Endocrinology
DGHO: German Society of Hematology and Medical Oncology
DGI: German Society of Infectiology
DGIM: German Society of Internal Medicine
DGMKG: German Society of Oral and Maxillofacial Surgery
DGN: German Society of Nuclear Medicine
DGU: German Society of Urology
DGVS: German Society of Gastoenterology, Digestive and Metabolic Diseases
DKG: German Cancer Society
DVPZ: Umbrella Organisation of Prostate Centres Germany
EBA: Early benefit assessment
EMA: European Medicines Agency
FEV1: Forced expiratory volume in 1 second
G-BA: Federal Joint Committee
GH: Growth hormone
GLSG: German Low Grade Lymphoma Study Group
Hb: Haemoglobin
HbA1c: Glycated haemoglobin
HIV: Human immunodeficiency virus
HRQoL: Health related quality of life
HTA: Health technology assessment
HZM: Helmholtz Centre Munich
IGF-1: Insulin-like growth factor 1
IQWiG: Institute for Quality and Efficiency in Healthcare
MCR: Major cytogenic response
mUFC: Median urinary free cortisol
NICE: National Institute for Health and Clinical Excellence
ORR: Objective response rate
OS: Overall survival
PEP: Primary endpoint
PFS: Progression-free survival
PM: Pharmaceutical manufacturer
POA: Working Group on Pulmonary Oncology
rPFS: Radiographic progression-free survival
SVR: Sustained viral response
ULN: Upper limit of normal
VR: Viral response
ZSE: Centre for Rare Diseases
