# Supplementary material for: Inconsistent approaches of the G-BA regarding acceptance of primary study endpoints as being relevant to patients - an analysis of three disease areas: oncological, metabolic, and infectious diseases
Source: BMC Health Serv Res. 2016 Nov 14;16:651. doi: 10.1186/s12913-016-1902-8 (PMC5109700; doi:10.1186/s12913-016-1902-8)
Supplement: Additional file 1: Table S1. — Rationale of categorisation of PEPs as symptomatic and asymptomatic. List of the rationale of categorisation as symptomatic and asymptomatic for all PEPs included in the analysis. (DOCX 14 kb) [file 12913_2016_1902_MOESM1_ESM.docx]

**Table S1: Rationale of categorisation of PEPs as symptomatic and asymptomatic.**

|  | **PEP** | **Basis of categorisation** |
| --- | --- | --- |
| **Symptomatic** | OS | Felt by patient |
|  | 6MWT | Felt by patient |
|  | Overall cure | Felt by patient |
|  | Complete durable tumour & symptomatic response | Definition (**symptomatic** response) |
|  | Partial durable tumour & symptomatic response | Definition (**symptomatic** response) |
|  | Reduction in spleen volume | G-BA oral hearing, statements by members of the ASIM, the DGVS and the European Gaucher Alliance |
|  | ≥35% reduction in spleen volume | G-BA oral hearing, statement of a member of the DGHO |
| **Asymptomatic** | Haematocrit control without phlebotomy | Laboratory parameter |
|  | MCR | Laboratory parameter |
|  | HbA1c | Laboratory parameter |
|  | Biochemical control (mean GH <2.5ug/L and normalisation of IGF-1) | Laboratory parameter |
|  | mUFC | Laboratory parameter |
|  | Haemoglobin level | Laboratory parameter |
|  | Thrombocyte count | Laboratory parameter |
|  | FEV1 | Laboratory parameter |
|  | Viral response (VR, SVR) | Laboratory parameter |
|  | PFS | Determined by imaging techniques, not based on symptoms, as per statements in the G-BA decision rationales |
|  | ORR | Determined by imaging techniques, as per statements in the G-BA decision rationales |
|  | Reduction in liver volume | G-BA oral hearing minutes, data from PM dossier |

6MWT: 6-minute walk test, ASIM: Working Group for Congenital Metabolic Disorders in Internal Medicine, DGHO: German Society of Hematology and Medical Oncology, DGVS: German Society of Gastroenterology, Digestive and Metabolic Diseases, FEV1: Forced expiratory volume in 1 second, G-BA: Federal Joint Committee, GH: Growth hormone, HbA1c: Glycated haemoglobin, IGF-1: Insulin-like growth factor 1, MCR: Major cytogenic response, mUFC: Median urinary free cortisol, ORR: Objective response rate, OS: Overall survival, PEP: Primary endpoint, PFS: Progression-free survival, PM: Pharmaceutical manufacturer, SVR: Sustained viral response, VR: Viral response.
